# Bilaterally Threaded, Minimal Invasive, Elastic Locking Intramedullary Nailing (ELIN) for the Treatment of Clavicle Fractures

**DOI:** 10.1111/os.12612

**Published:** 2020-02-19

**Authors:** Kifayat Ullah, Saima Khan, Yong‐Qing Wang, Zhi‐Hui Zhao, Peng Cheng, Basanta Sapkota, Liang Ren, Samiullah Khan, Mujeeb Ur Rehman, Yuan Xue

**Affiliations:** ^1^ Department of Orthopedic Surgery, Tianjin Fourth Central Hospital Tianjin Medical University Tianjin China; ^2^ Department of Infertility and Reproductive Endocrinology Tianjin Medical University Central Hospital of Obstetrics and Gynecology Tianjin China; ^3^ Western Hospital PVT. LTD Nepalgunj Nepal; ^4^ Department of Gastroenterology and Hepatology Tianjin Medical University General Hospital Tianjin China; ^5^ Department of Cardiovascular and Thoracic Surgery Tianjin Medical University General Hospital Tianjin China; ^6^ Department of Orthopedic Surgery Tianjin Medical University General Hospital Tianjin China

**Keywords:** Clavicle, Fractures, Intramedullary nail, Locking, Minimal invasive

## Abstract

**Objective:**

To evaluate and present the effectiveness of this innovatively designed, elastic locking intramedullary nail (ELIN) in fixation of clavicle fractures.

**Methods:**

The study included 38 patients from July 2014 to July 2017. All of them received intramedullary fixation treated with ELIN, 22 were males and 16 females. The mean age of the patients was 54 years. There were twenty right‐side and 18 left‐side clavicular fractures. Radiographs were taken to assess the fracture type: 21 were type A, 16 type B, and one type C. General anesthesia or cervical block was given to all patients. A small incision of 3–5 cm was given only to those who needed mini‐open reduction. The administration of ELIN and reduction of the fracture was made sure with a C arm machine. After a follow‐up of 8 to 33 months, the clinical outcomes were assessed and evaluated. The constant scores and disabilities of the arm, shoulder and hand questionnaire (DASH) were used to determine the outcomes and functional status of the patients. The study was done accordingly to the guidelines provided by the ethics committee.

**Results:**

Mean operation time was 25.63 min. Mean follow‐up time was 16.5 months. The rate of closed reduction and open reduction was 84% and 16% respectively. There was no shortening of the clavicle. There was no breakage of the nail, though bending of the nail occurred in one patient. Superficial skin infection occurred in three patients at insertion points or the nail tip which was embedded subcutaneously. Skin erosion with nail exposure occurred in a patient with no significant infection. All the other patients had excellent shoulder function. A mini scar was observed in seven patients all the other patients had no scar. Asymmetry was observed in three patients. The mean Constant score was 98.47 and the mean DASH score was 1.55 at the last follow‐up. The implant was removed in all the patients.

**Conclusion:**

Clavicular fractures treated with ELIN is minimally invasive, which presents a safe and novel surgical technique with less complications and a high success rate, excellent aesthetic and quick recovery after surgery. ELIN restores the micro‐dynamic stress at the fracture ends and promotes fracture healing, keeps intact the fracture hematoma and maintains the blood supply, accelerates healing and thus leads to faster osseous healing and better restoration of clavicle length.

## Introduction

The clavicle is considered one of the most commonly fractured bones, and accounts for 2.6%–12% of all fracture. Middle third fractures accounts for 80%, whereas fractures of the lateral and medial third of the clavicle account for 15% and 5% of all fractures, respectively^1^. According to the Allman classification, fractures of the clavicle are divided into three groups: (i) fractures of the middle third; (ii) fractures of the lateral third; and (iii) fractures of the medial third^2^. The incidence of clavicle fractures is high in young athletes, and a direct fall on the shoulder is the most common cause of this injury^3^. The surgical indications for mid‐shaft clavicle fractures are controversial and have changed recently. Traditionally, most of the clavicle fractures have been treated conservatively. There is a general agreement that un‐displaced fractures should be treated non‐operatively^4^. Some studies even suggest that displaced mid‐shaft clavicle fractures should be treated non‐operatively, and they believe that non‐operative treatment yields good results without incurring the potential complications of surgery^5^. However, recent studies reported poorer results following non‐operative treatment; displaced fractures treated conservatively heal with some degree of shortening and therefore result in malunion unless treated operatively. Malunion can become symptomatic with pain, loss of strength, rapid fatigue, numbness or paresthesia of the arm and hand, as well as cosmetic complaints^6^, ^7^. Due to poor results of non‐operative treatment, some literature now recommends open reduction and internal fixation of displaced fractures of the clavicle^8^. Currently, there is no debate on surgical treatment modalities for displaced mid‐shaft clavicle fractures. The only way to prevent a malunion or nonunion in a dislocated mid‐shaft clavicle fracture is an open reduction with internal fixation or a percutaneous procedure – the most commonly used are plate fixation or intramedullary nail (IMN) fixation^9^, ^10^. Some studies indicate no difference in functional outcomes or complications after plate fixation or intramedullary fixation for displaced mid‐shaft clavicle fractures^11^, ^12^, ^13^. However, a randomized clinical trial supports primary plate fixation of completely displaced mid‐shaft clavicular fractures^14^. In general, some scholars regard the plate fixation as a standard operative treatment for clavicle fractures^15^, ^16^, ^17^, ^18^, ^19^. However, plate fixation advantages are compromised by large skin incision, extensive soft tissue dissection that potentially results in damage to the superior clavicular nerves and subsequent parenthesis, implant prominence, infection, scarring, hardware failure and re‐fracture after implant removal, and mostly the patients have aesthetic complaints^20^, ^21^.

Due to tremendous advancement in intramedullary fixation methods and devices, its low complication rates, minimally invasive approach, short operation time and quick recovery, intramedullary fixation has become a popular and preferable choice of treatment compared to plate fixation and conservative treatment. Studies^21^ suggested that intramedullary fixation is a more advantageous method for the treatment of mid‐shaft clavicle fractures, achieving excellent aesthetic and early recovery after surgery. For intramedullary fixation, the most common devices in clinics used are Knowles pinning^22^, ^23^, elastic stable titanium intramedullary nailing^24^, ^25^, Rockwood clavicle pin^26^, Acumed clavicle rod^27^, Hagie pin^28^, expandable elastic locking intramedullary nail^29^ and threaded elastic intramedullary nail^30^. All these devices have their own merits and demerits. Due to minimally invasive, low complication rates and high patient satisfaction, locked clavicle intramedullary nails have become popular and most acceptable to surgeons^29^, ^30^.

This study describes the technique of minimally invasive intramedullary fixation of midclavicular fractures with ELIN. The aims of this study were: (i) to assess the experience of this new device with its effectiveness, functional outcomes, cosmetic and minimally invasive aspects; (ii) to evaluate its faster recovery, keeping the anatomical or original shape of the clavicle; and (iii) to determine and present ELIN is an alternative treatment for clavicular fractures.

## Materials and Methods

### *Inclusion Criteria*

Inclusion criteria for this study were: (i) displaced middle 1/3 OTA type A, B and C, lateral 1/3 and medial 1/3 OTA type A and B clavicular fractures; (ii) patient treated with ELIN; (iii) patient aged between 12 to 85 years; and (iv) follow‐up record from 8 to 33 months.

### *Exclusion Criteria*

Exclusion criteria for this study were: (i) shortening of the clavicle by more than 2 cm; (ii) associated injuries and fractures; (iii) pathological fractures; (iv) open fracture; and (v) neurovascular injury.

### *Patients*

This study included patients at our hospital from July 2014 to July 2017. They were all retrospectively reviewed. All the patients were diagnosed with displaced mid‐shaft clavicular fractures, medial 1/3, lateral 1/3, within a week and treated with ELIN fixation. Total of 38 patients were included, 22 males and 16 females. The mean age of the patients was 54 years. There were 20 right‐side clavicular fractures and 18 left‐side clavicular fractures. The causes of the injury (Table 1) included sports/athletics (n = 12), fall on surface board (n = 2), car accident (n = 5), motorbike (n = 6), bicycle (n = 4), horse fall (n = 2), direct hit (n = 2), fall from a tree (n = 1), fall from balcony (n = 1), squeeze against a wall by a trailer (n = 1), and stairs fall (n = 2). After admittance, radiographs were taken to assess the fracture type and post‐traumatic clavicular shortening. The fractures were classified according to the Orthopaedic Trauma Association (OTA) classification system^31^ for mid‐, lateral‐ and medial clavicular fractures. Twenty‐one were type A, 16 were type B, and one was type C.

**Table 1:** Cause of injury

No	Cause of injury	Total patients
1	Sports/ Athletics	12
2	Fall on a surface board	2
3	Car accident	5
4	Motorbike	6
5	Bicycle	4
6	Horse Fall	2
7	Direct hit	2
8	Fall from a tree	1
9	Fall from balcony	1
10	Squeeze against a wall by a trailer	1
11	Stairs fall	2

Patients who were found to be eligible for this study were informed about the study protocol. Afterwards, a written informed consent was obtained. This study was approved by the ethics committee of our institution, and was done according to the guidelines provided by the ethics committee.

### *Materials*

**Figure 1:**
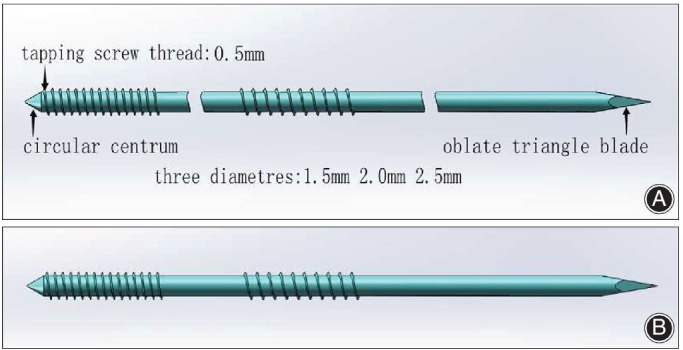
(A) Simulation design diagram of Elastic, Locking, Intramedullary Nail. (B) Patent ELIN.

The ELIN is composed of titanium alloy (Tc4), titanium plus nickel alloy and stainless steel (317L).The ELIN was made in Kang Li Min Medical Devices Co. Ltd., Tianjin, China. The design of the nail is that there are two tapping screw threads, one at the proximal end and another one at the distal end with a sharp tail. The length of the nail is 250 mm with different diameters, including 1.5 mm, 2 mm, and 2.5 mm (Fig. 1).

### *Operative Procedure*

#### *Anesthesia*

General anesthesia or cervical block was given.

#### *Position*

The patient was in supine position. To maintain a 30° angle space between the affected shoulder and the patient, a 6–12 cm pillar‐shaped pillow was put under the affected shoulder. The surgeries were executed by two to three surgeons.

#### *Sterilization*

Proper sterilization of the skin above the fracture site was done, to ensure proper disinfection, the sterilization was widened to the lower level of the breast, neck, shoulder, upper arm and above the scapula.

#### *ELIN Administration*

**Figure 2:**
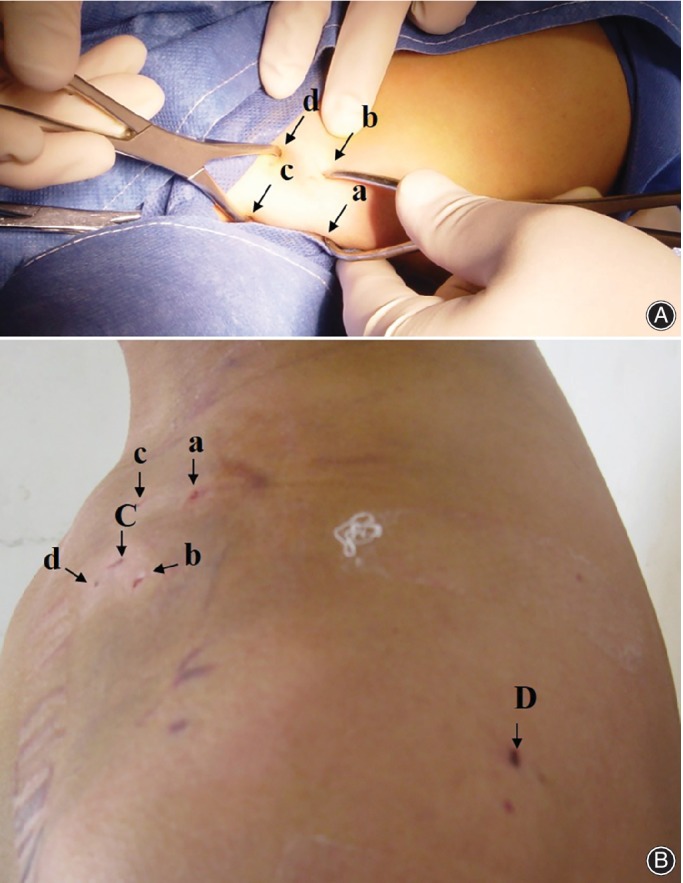
(A) a, b, c, d denotes insertion of the two weber clamps while doing the reduction and insertion of ELIN. (B) a, b, c, d denotes insertion points of the two weber clamps, C denotes antegrade insertion point of ELIN whereas D denotes the retrograde insertion point (during the procedure) and pull out point of ELIN (after the complete healing of the fracture).

Position of the fracture was marked. To avoid damaging tissues and organs, two pointed‐reduction clamps (weber clamps) were slid down close to the cortex through the skin near the fracture site. The medial and lateral fractured ends were held by the weber clamps (Fig. 2). Primarily, the lateral end was elevated then the fracture site was located through the skin using an awl. A suitable ELIN ranging 1.5 to 2.5 mm in diameters according to the size of the medullary cavity of the clavicle was selected, attached to the drill and drilled under the C‐arm radiographic control until it came out at acromial end at clavicular tubercle treating medial 1/3 and middle 1/3 fractures, whereas, at acromion process of the scapula for lateral 1/3. Then the drill was attached to the lateral threaded end of ELIN which was drilled out while keeping the medial threaded end of ELIN at the level of the lateral fracture end. Secondarily, the medial fracture end was elevated, and the fracture site was located using an awl. Then, reduction and fracture alignment was done. Medial and lateral fracture ends were held together tightly by two weber clamps, single and displaced fragments were preserved *in situ*, with soft tissue connection. ELIN was drilled into the medial fracture end. Weber clamps were removed. Reduction of the fracture and entrance of ELIN to the medial fracture end was made sure by C‐arm.

#### *ELIN's Position*

Treating middle 1/3 ELIN was drilled in until it reached about 20–40 mm away from the medial clavicular end, treating medial 1/3 ELIN was drilled into the sternum while treating lateral 1/3 ELIN was drilled in until it reached the mid‐shaft of the clavicle. At last ELIN was cut at lateral end leaving 5 mm tip out of the skin. The tip was bent and embedded subcutaneously.

#### *Closed vs Open Reduction*

**Figure 3:**
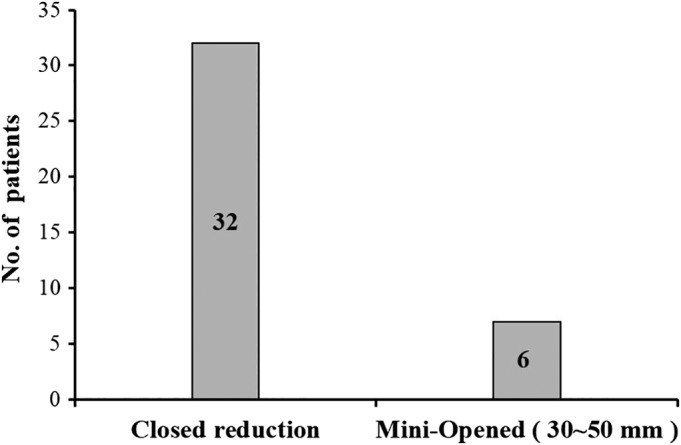
A Graph showing the Ratio of closed reduction (no incision) and Mini‐opened (small incision 30 mm to 50 mm) in our study.

The rate of closed reduction was 84% and open reduction was 16% (Fig. 3). A small incision of 3‐5 cm was made for all the other patients who needed mini‐open reduction for the reason of fat, severely displaced fractures contained fragmentary displacements, or osteoporotic fractures.

#### *Postoperative Rehabilitation*

A shoulder sling was applied to all the patients for 3 weeks aiming to support the affected limb. The patients were asked to do passive non‐weight bearing movement and exercises, initiated in a tolerable arc movement the next day after the operation. As the range of motion of shoulder increased during the 3 weeks after the surgery, shoulder strengthening exercise were initiated.

### *Outcome Measures*

#### *Radiographic Evaluation*

After the clinical assessment of the clavicle and shoulder, anteroposterior (AP) X‐ray examination of the injured shoulder was done. The result of surgery was also determined with radiographic examination. A standard X‐ray (AP view) for the clavicle was used. The clavicle length was evaluated using computed tomography (CT). Shortening of the clavicle was indicated by radiography and comparison to the intact opposite side by comparing the distance between two fixed bony landmarks such as the medial end of the clavicle and the acromioclavicular joint.

#### *Asymmetry of the Shoulder*

The cosmetic outcomes were determined with special regard to asymmetry of the shoulder, a visible deformity caused by callus hump or hypertrophic scars. Asymmetry of the shoulder was determined by measuring the distance from the center of the jugular fossa to the lateral tip of the acromion. In comparison with the intact contralateral side, a difference greater than 0.5 cm was considered significant asymmetry.

#### *DASH and Constant Score*

The Constant score^32^ and disabilities of the arm, shoulder and hand questionnaire (DASH) score^33^ were used. The aim of the DASH score was to evaluate the level of disability of the shoulder and arm. The DASH score ranges from 0 (disability) to 100 (sever disability). The Constant score is a 100 points scale and the aim of using this score was to measure the pain, activities of daily living, strength and range of motion of the shoulder joint. Grading of the Constant score is excellent (86–100), good (71–85), fair (56–70), and poor (<56).

#### *Statistical Analysis*

When normally distributed, quantitative variables were presented as the median and standard deviations and analyzed using Student's *t*‐test. For non‐normally distributed variables, quantitative data was presented as the medians with interquartile ranges and analyzed using the Wilcoxon rank sum test. Enumeration variables were presented as absolute numbers with percentages, and analyzed using the chi‐square test and Fisher's exact test. Significance was set at *P* < 0.05. All the analyses were completed using the SPSS 17.0 Windows (SPSS Inc., Chicago, IL, USA) statistics program.

## Results

### *Operation and Follow‐up Time*

All the operations were done from 15 to 40 min; the mean operation time was 25.7 min. The mean fluoroscopy time was 3 min. All the patients were given antibiotics; local skin dressing was done one to two times. All the patients were followed up by half month, 1, 2, 3, 6, and 12 months. No patient was lost to the follow‐up. All the patients were followed‐up postoperatively for a minimum period of 8 months. The mean follow‐up time was 16.5 months (Table A1), and the rate of closed reduction was 84%.

### *ELIN Complications*

Regards to complications, no patient suffered from neuromuscular compromise, impairment, deep infection, pulmonary injuries, shortening, re‐fracture or nonunion. Three patients with superficial infection at the site of the nail tip or insertion point that was embedded subcutaneously were treated with wound debridement and antibiotic therapy. Skin erosion with nail exposure occurred in one patient; however, there was no significant infection, and healed without any other intervention.

Regarding the medial migration of the nail, no medial migration of the nail occurred in our study. The pressure sores at the lateral end occurred in eight patients (21%). There was no breakage of nail, one nail bending occurred 20 mm away from the acromial joint. All fractures healed, no malunion or nonunion was observed. No revision surgery was required for any patient. No shortening of the clavicle was observed (Table A2).

### *Asymmetry of the Shoulder*

Regarding the asymmetry, the distance from the center of the jugular fossa to the lateral tip of the acromion was measured and a difference greater than 0.5 cm was regarded as asymmetry. Asymmetry was observed in only three (7.89%) patients (Table A2).

### *DASH and Constant Score*

Two patients complained of functional limitation of the shoulder joint. Three patients had the nail protrusion and pain at the lateral end without any obvious complications. The DASH score was excellent in (n = 35) and good in (n = 3). No disability of the shoulder and arm was observed. The mean DASH score was 1.55, and mean constant score was 98.47 (Table A2).

Constant score was excellent in (n = 37), good (n = 1), fair (n = 0), and poor (n = 0) patients. Excellent strength, less pain, and satisfactory daily life activities with great range of motion were achieved in all patients.

### *Cosmetic Outcomes*

Cosmetic outcomes were assessed with regards to asymmetry and/or an obvious callus hump or scar. An obvious skin scar in two patients was observed at the incision point and a mini scar in five patients. All the other patients had no scar.

### *Implant Removal*

**Figure 4:**
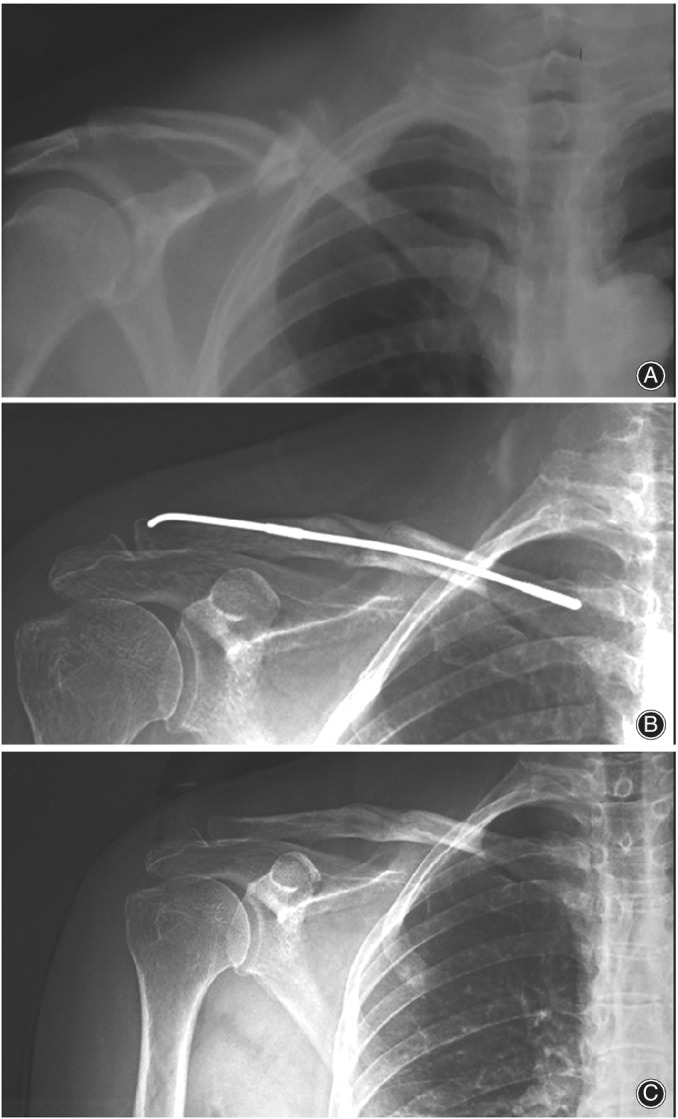
(A) Preoperative x‐ray showing a patient with mid clavicular fracture. (B) x‐ray showing midclavicular fracture treated with ELIN. (C) x‐ray showing the complete healing of the fracture after the removal of ELIN.

**Figure 5:**
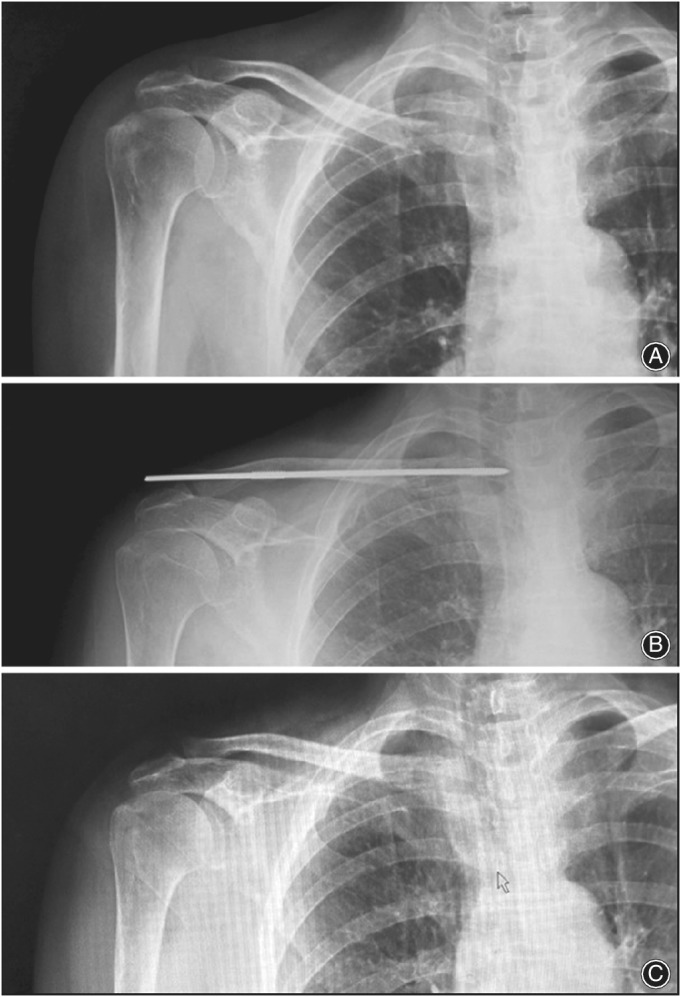
(A) Preoperative x‐ray showing of a patient with medial 1/3 clavicular fracture preoperative. (B) postoperative x‐ray showing medial 1/3 fracture treated with ELIN. (C) x‐ray showing the complete healing of the fracture after the removal of ELIN.

**Figure 6:**
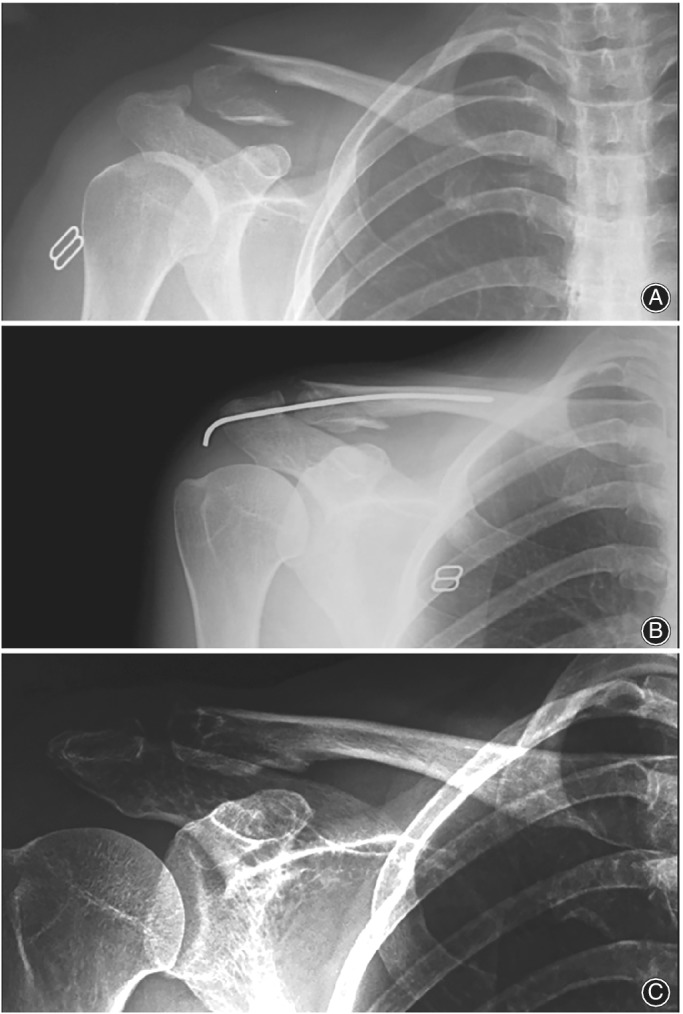
(A) Preoperative x‐ray showing a patient with lateral 1/3 clavicular fracture. (B) postoperative x‐ray showing lateral 1/3 fracture treated with ELIN. (C) x‐ray showing the complete healing of the fracture after the removal of ELIN.

**Figure 7:**
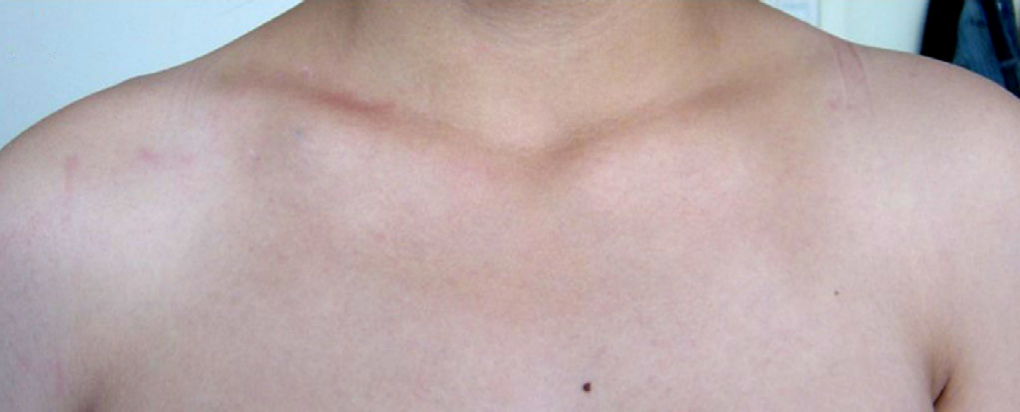
Cosmetic results after the removal of ELIN in a male patient aged 30 with middle 1/3 clavicle fracture.

The implant was removed at a mean of 13 (12–18) weeks postoperatively (Figs 4, 5, 6, 7). After examining the X‐rays and making sure that the fractures had healed, the patients were informed that the nail would be removed in the outpatient departments (OPD) through local anesthesia. There was no need for second time hospitalization for the removal of the implant. A small incision was given under local anesthesia above the lateral nail tip, the nail tip was exposed and pulled out with the help of needle‐holding forceps in anticlockwise manner. ELIN was removed in all the patients. All the fractures healed in 2 to 3 months after surgery, no nonunion occurred in our study.

General information, fracture type, operative information, follow‐up and implant removal time of the patients treated with ELIN is given in Table A1 in the Appendix and Complications sections; functional outcomes and cosmetic outcomes of the patients treated with ELIN is given in Table A2 in the Appendix.

## Discussion

The clavicle is the only bone that connects the upper limb to the trunk. The clavicle functions as a strut. The medial end and the lateral end have flat expansions linked by tubular middle which has sparse medullary cavity. Its position is prominent, prone to be fractured by fall or violence. The middle one‐third is the most vulnerable area to fracture^34^, ^35^. There are two types of treatment for clavicle fractures: non‐operative and operative treatment. Studies have shown that the conservative treatment for clavicle fractures in not ideal, with about 10%–30% resulting in poor recovery, shortening, irregular fractures, line angulation, even deformity healing and poor shoulder function. The clavicle, as an important part displaying the beauty, and is always the main focus of surgeons to retain its original length and appearance to the greatest extent. Therefore, most of the surgeons prefers surgical treatment in clinical practice^24^, ^36^. The surgical treatment for clavicular fracture includes plate fixation and intramedullary nail fixation. The effect of surgical treatment is more satisfactory than conservative treatment. However, the high incidence of complications after plate fixation is troubling, and the failure cases have been reported frequently^20^, ^21^. Compared with plate fixation, intramedullary nail has shorter operative time and lower risk of re‐fracture, but there is no significant difference in functional recovery, superficial infection, transient brachial plexus injury, nonunion, delayed union, internal fixation failure and revision^21^, ^37^. Smith *et al*.^38^ reports that after removal of the locked compression plate, clavicle strength was reduced when subjected to binding movements, resulting fracturing through the vacant screw holes. Thus, IM fixation has an advantage over plate fixation in terms of the lower risk of re‐fracture in the immediate postoperative period if implant removal is performed. In recent years, with the rapid development of intramedullary nail technology, intramedullary nail fixation for clavicle fractures has achieved good and satisfactory results. The application of elastic locking intramedullary nail (ELIN) in the treatment of clavicular fracture, compared with steel plate, has a shorter operation time, the incision is small, fracture healing is quick, and the infection rate is low^9^, ^10^.

The medial fragment of the clavicle is elevated by the clavicular head of the sternocleidomastoid muscle, which attaches onto the posterior aspect of the medial portion of the clavicle. The pectoralis major contributes to adduction and inward rotation of the shoulder^39^. The force due to the sternocleidomastoid muscle at the proximal end of the clavicle is upward and backward, whereas the force at armorial end, due to pectoralis major muscle and pull of the arm, is down and backward. These two forces in different directions cause the rotation of the clavicle. This torsion of the clavicle often causes spiral, oblique or comminuted fracture. Bad reduction of the clavicle fractures not only affects its appearance, but also affects the load‐bearing capacity of the upper limb^40^. According to Wolff's law, there is a certain amount of stress stimulation at the broken ends of the clavicle after fracture, and it does not damage the new blood vessels, which is conducive to the formation of callus and promotes fracture healing^41^. The basic principle of ELIN is EO (elastic osteosynthesis). ELIN is elastic adjusted centrally and firmly fixed to the S‐shape of the clavicle. ELIN conforms to the principle of biomechanics of the clavicle. As it is bilaterally threaded, due to its locking quality it does not let the clavicle twist or rotate, thus balancing two forces of the clavicle in different directions. From mechanical point of view, ELIN produces early elastic fixation at the end of the clavicle fracture which advocate stress shielding at the fracture point. Due to this sustained stress‐shielding mechanism and with the movement of the shoulder joint, micro‐dynamic movements at the fracture point help to promote callus formation, thus accelerating fracture healing. In addition, the minimally invasive technology is the reason for accelerating fracture healing, which protects the accumulation of blood at the fracture ends from being removed. The accumulation of blood at the fracture site contains bone marrow stem cells, which are conducive to fracture healing. Thus, minimally invasive ELIN preserves the fracture hematoma at the fractures ends and accelerates healing.

Kirschner wires (K‐wires) were the first IM form used for clavicle fractures. In a review study of 47 patients, Lyons and Rockwood reported eight implant‐related deaths, along with implant migration to the heart, aorta, lungs, and cervical spine. This study indicates high‐risk complications regarding the use of K‐wires, and relegate their use in the treatment of clavicle fractures^42^. No migration of ELIN occurred in our study. Hagie pins and Rockwood pins are also used for the treatment of mid‐shaft clavicle fractures, but Mudd *et al*.^26^ and Strauss *et al*.^28^ recommended against the continued use of these implants, given the high rate of complications associated with their use. Among the 18 patients in their study, complications occurred in 14 patients. Lateral implant pain and posterior skin erosion are the most common – and potentially morbid – complications, and these occur at an unacceptable rate. Eichinger *et al*.^43^ agrees with Mudd and Strauss, and believes that the use of alternative implants should be considered if IM fixation is planned. Compared to Hagie pins and Rockwood pins, lateral implant pain and posterior skin erosion was considerably lower in our study with ELIN. Jubel and Kettler^24^, ^44^ successfully used titanium elastic intramedullary nail in the treatment of clavicle fractures. They think that elastic intramedullary nail, as a minimally invasive treatment technology for middle clavicle fracture, can guarantee good functional recovery postoperatively. But complications such as nonunion, implant related problems and shortness have been reported in the literature. Frigg *et al*. reported a 70% complication rate, including a revision rate of 36% out of the 34 patients treated with titanium elastic nail (TEN)^45^. In a study Rollo G reported a nonunion rate of 0.1% and 15%, he further added that there are several predisposing factors for the onset of complications, general factors connected with the patients and specific factors related to the fracture site. The purpose of Rollo's study was to review the etiology of nonunion of the clavicle in its atrophic form and investigate the outcomes of the revision treatment in a single step. Rollo also reported that the fibula splint, tricortical bone graft, autologous bone graft and iliac crest bone graft all have mechanical and strong biological values to quickly heal the nonunion^46^, ^47^. On the other hand all patients treated with ELIN had smooth healing with less complications and more satisfactory results with no revision required for any patient. ELIN reduces the chances of infection and thus nonunion. Yong‐Qing Wang^30^ used TEIN to treat midclavicular fracture by closed reduction and internal fixation. Its clinical application when compared with the steel plate and Kirschner wire, TEIN had the advantages of low trauma, good blood supply, less stress shielding, and reliable and simple operation. TEIN has good biocompatibility, good anti fatigue, corrosion resistance, wear resistance, and provide safe stability. However, its problems such as low anti‐rotation capacity, its propensity to emerge from the clavicular end, and poor effect for treatment of comminuted clavicle fractures cannot be ignored. Meanwhile, ELIN is bilaterally threaded elastic with a locking function. Threads at both the ends of the nail holds the two fragments tight. It can resist rotation, shortening and compression of the fracture. Many intramedullary nails were used in the past for the treatment of mid‐shaft clavicular fractures but with many complications. Thus, to overcome these complications and treat most of the clavicular fractures (medial 1/3, middle 1/3 and lateral 1/3) this innovative ELIN was designed and used in our study.

**Figure 8:**
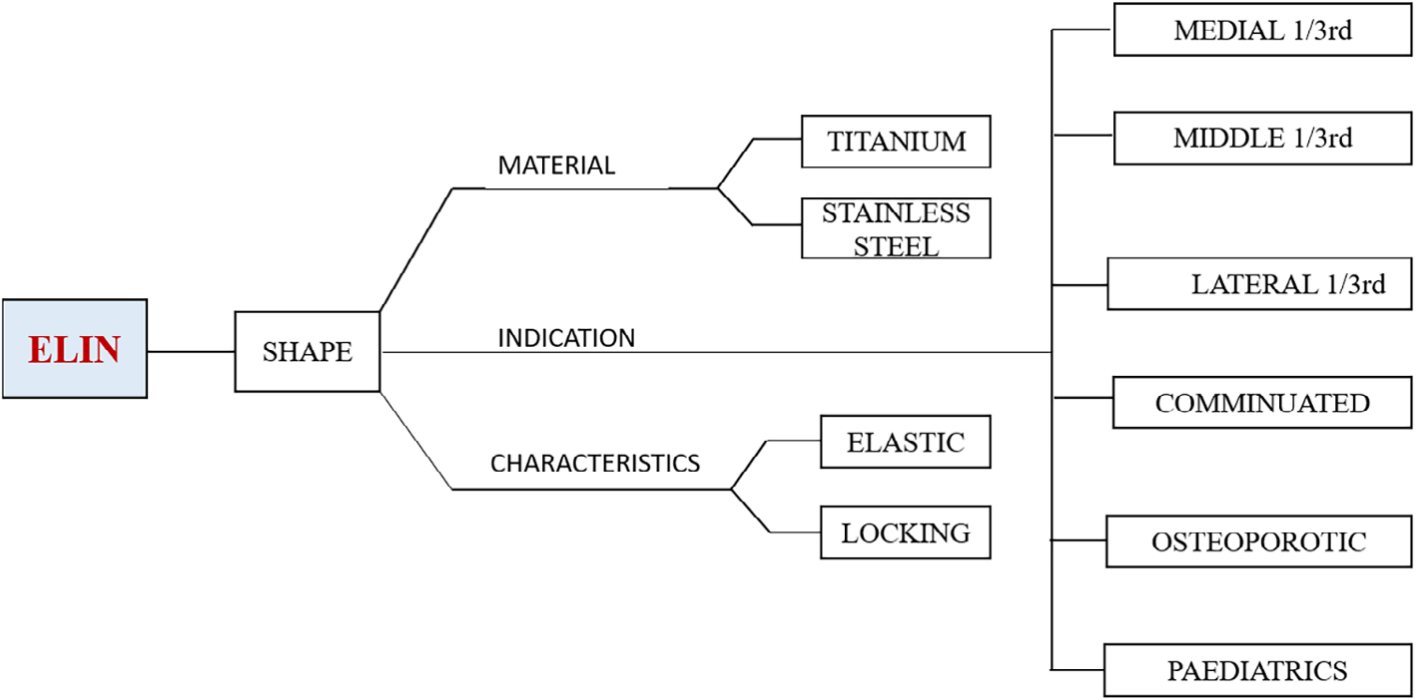
Schematic summary of ELIN.

Clavicular fracture fixation with ELIN is a minimally invasive operation (Fig. 8). It is a new direction in the development of Orthopaedics. Compared with other surgical methods, ELIN has advantages: it is elastic and can adapt easily to medullary cavity, and its bilateral‐threading locking function holds the fractured ends tightly together. Because of these characteristics it is suitable for the treatment of most clavicular fractures. There is no scar (thus preserving appearances), no need for second time hospitalization and secondary operation for its removal, less pain, and the implant can be easily pulled and removed at OPD with a simple procedure from the acromial end embedded under the skin thus reducing the chances of injury and infection. Because of its locking quality, it is firmly fixed within the clavicular medullary cavity and cannot be emerged at the clavicular end, having less chances of skin irritation. The operation time is short, the instruments used are simple. The price is low, quality is good and it is easily available. Closed reduction and internal fixation with ELIN must be carried out under the guidance of the “C” arm X‐ray machine. Patients and doctors are subjected to high dose X‐ray radiation. Especially patients with severe obesity, local swelling and relatively difficult reduction suffer longer radiation time.

Other IMN devices are mostly focused on one type of clavicular fracture, while in our study we have used ELIN for all types of clavicular fractures – middle 1/3, medial 1/3 fractures, lateral 1/3, type c fractures, fractures in children, and osteoporotic fractures. It is worth mentioning here that we use only one‐side threaded nail while treating lateral 1/3 fractures, as the lateral thread comes either at the fracture line, causing more chances of re‐fracture, or at the clavicular acromial joint, making it static with no movement or restricted movement postoperatively causing more chances of bending and breakage of the nail.

After 8 weeks, ELIN fixation fractures healed firmly and there was no loosening or breakage of the nail. ELIN retains the integrity of the periosteum, soft tissue and blood vessels around the fracture site. ELIN fixation requires no incision or, rarely, small incision ( i.e. 3–5 cm) which maximizes the limit to preserve the appearance of the local body surface.

The effect of ELIN as a minimally invasive treatment for clavicular fractures is related to the type of fracture and the degree of osteoporosis. ELIN for the treatment of middle 1/3 fractures with good bone quality is the most effective. For comminuted fractures and osteoporotic bone, ELIN with long threads should be used bcause ELIN with long thread has strong resistance to contraction and shortening. But for severe osteoporotic patients, the reduction and fixation effect is poor. In our data, we have a case of an 85‐year‐old woman with severe osteoporosis; two months after the surgery, her shoulder joint activity was good. But due to the rotation of the ELIN, the nail tip at the acromial end was slightly protruded, piercing the skin. Three months postoperatively the fracture healed completely and ELIN was removed, the functional recovery was satisfactory. ELIN should be used cautiously for osteoporotic patients. ELIN is firmly fixed and locked by trabecular bone in the medullary cavity. Osteoporosis will lead to poor fixation, reduction, rotation and protrusion of the nail.

This study has some limitations, including a small number of patients, poor results in osteoporotic patients, and a cautionary use of ELIN for type c fractures. Also, this is a single center study; a randomized controlled study with large population is needed in multiple centers to further assess the clinical outcomes of ELIN. We also hold the opinion that, besides clavicular fractures, ELIN can be used for factures of other tubular bones such as fibula, phalanges, radius and ulna; however, these uses of ELIN need further study, research, changes in diameter and length of the ELIN.

### *Conclusions*

Minimally invasive surgery with intramedullary nailing is a preferred option for patients with clavicle fracture and doctors alike. With the improvement and advancement of living standards, the requirements of a quick recovery of injured limbs and satisfactory function are also increasing. The patients are obviously more willing to accept some trauma, quick recovery, a small amount of scarring or no scar. We believe that ELIN is a minimally invasive and novel treatment technique. ELIN's elastic osteosynthesis (EO) conforms to the shape of the medullary cavity of the clavicle, restores the micro‐dynamic stress at the fracture ends and promotes fracture healing. With only a small degree of trauma with no scar or a minor scar, it maintains the natural appearance of the body surface making it a simple operation with great therapeutic effect for clavicular fractures. ELIN can be an alternative for the treatment of almost all types of clavicle fractures with less complications, quick recovery and good cosmetic results.

## Declarations

### *Ethics approval and Consent to participate*

Informed consent was obtained from patients, which covered the publication of all the personal and medical details that have been included in the manuscript, including images. This study was approved by the ethical review board of our institution.

### *Disclosure*

The authors declare that they have no conflict of interest.

### *Ethical approval*

This study was approved by the ethical committee board of Tianjin 4th Central Hospital, Tianjin Medical University.

### *Informed consent*

Informed consent was obtained from all individual participants included in the study.

## Appendix

**Table A1:** General information, fracture type, operative information, follow‐up and implant removal time of the patients treated with ELIN (n = 38)

Number	Age	Sex	Side	Fracture type	OTA Classification	Incision (mm)	Operation time(min)	Blood loss (mL)	Closed reduction	Follow up months	Implants removal time (weeks)
1	61	Male	Right	Lateral 1/3	A	N	15	10	Y	23	13
2	56	Male	Left	Middle 1/3rd	A	N	30	12	Y	12	13
3	68	Female	Right	Lateral 1/3	A	N	25	11	Y	8	10
4	56	Female	Right	Middle 1/3rd	A	N	35	8	Y	13	13
5	46	Female	Left	Middle 1/3rd	B	N	20	10	Y	24	15
6	44	Male	Right	Medial 1/3rd	B	N	30	10	Y	32	12
7	41	Male	Left	Middle 1/3rd	B	30	15	12	N	16	13
8	63	Male	Right	Lateral 1/3	B	N	30	13	Y	12	10
9	71	Female	Left	Middle 1/3rd	A	50	40	25	N	8	10
10	63	Male	Left	Middle 1/3rd	A	N	30	10	Y	17	13
11	48	Male	Right	Medial 1/3rd	A	N	35	14	Y	15	18
12	41	Male	Right	Middle 1/3rd	B	N	25	12	Y	26	10
13	46	Male	Right	Middle 1/3rd	B	N	20	10	Y	19	15
14	12	Male	Right	Middle 1/3rd	A	N	20	15	Y	9	13
15	36	Male	Right	Middle 1/3rd	C	40	25	20	N	33	18
16	57	Male	Right	Lateral 1/3	A	N	25	13	Y	9	13
17	73	Male	Left	Middle 1/3rd	A	40	30	16	N	10	13
18	64	Male	Right	Middle 1/3rd	A	N	20	12	Y	20	14
19	62	Female	Left	Middle 1/3rd	A	N	15	8	Y	12	16
20	19	Male	Left	Medial 1/3rd	A	N	24	10	Y	12	16
21	59	Female	Right	Middle 1/3rd	B	40	32	18	N	23	13
22	48	Male	Right	Middle 1/3rd	B	N	34	12	Y	25	12
23	54	Male	Left	Middle 1/3rd	A	N	18	8	Y	14	10
24	60	Male	Right	Middle 1/3rd	A	N	24	9	Y	18	13
25	57	Male	Left	Middle 1/3rd	B	N	22	7	Y	13	10
26	56	Female	Right	Middle 1/3rd	B	N	18	8	Y	24	12
27	61	Male	Left	Middle 1/3rd	B	N	16	8	Y	12	15
28	66	Female	Left	Middle 1/3rd	A	N	22	10	Y	8	18
29	62	Male	Left	Middle 1/3rd	B	N	28	18	Y	11	14
30	67	Female	Right	Middle 1/3rd	A	N	32	16	Y	16	16
31	30	Male	Left	Middle 1/3rd	B	N	24	12	Y	12	15
32	61	Female	Left	Middle 1/3rd	C	50	38	20	N	15	17
33	58	Female	Left	Middle 1/3rd	A	N	32	18	Y	19	12
34	45	Female	Right	Medial 1/3rd	A	N	24	16	Y	14	13
35	53	Female	Right	Middle 1/3rd	B	N	20	10	Y	15	15
36	52	Female	Right	Middle 1/3rd	A	40	26	20	N	23	16
37	51	Female	Right	Middle 1/3rd	B	N	25	17	Y	19	14
38	85	Female	Left	Middle 1/3rd	B	N	30	15	Y	16	18
AVG	54					41.42857143	25.63157895	12.97368421		16.5	13.71052632
SD	13.94160755					6.38876565	6.474326027	4.220793403		6.315186375	2.372073208

Footnotes: Y = Yes, N = No, OTA = Orthopedic Trauma Association.

**Table A2:** Complications, functional outcomes, and cosmetic outcomes of the patients treated with ELIN (n = 38)

Number	Constant score	DASH Score	Comparison with opposite side	Shortening	Complications	(Scar/No Scar)
1	100	0	Symmetrical	N	None	No Scar
2	100	0	Symmetrical	N	Pressure sores	No Scar
3	100	0	Symmetrical	N	None	No Scar
4	100	1	Symmetrical	N	None	No Scar
5	100	5	Symmetrical	N	Skin irritation/protrusion	No Scar
6	100	0	Symmetrical	N	None	No Scar
7	100	0	Non‐Symmetrical	Y	Pressure sores /Functional Limitation of the shoulder joint	Scar
8	100	0.8	Symmetrical	N	None	No Scar
9	100	0	Symmetrical	N	None	Scar
10	100	0.9	Symmetrical	N	None	No Scar
11	100	0	Symmetrical	N	Pressure sores / protrusion	No Scar
12	100	0	Symmetrical	N	None	No Scar
13	100	0	Symmetrical	N	None	No Scar
14	100	0	Symmetrical	N	Pressure sores / local infection	No Scar
15	100	0	Symmetrical	N	None	Scar
16	100	0.8	Symmetrical	N	None	No Scar
17	97	3.8	Symmetrical	N	None	Scar
18	100	0	Symmetrical	N	None	No Scar
19	96	4.2	Symmetrical	N	None	No Scar
20	97	2	Symmetrical	N	None	No Scar
21	78	5.8	Non‐Symmetrical	Y	Nail bending	Scar
22	100	0	Symmetrical	N	None	No Scar
23	95	3.8	Symmetrical	N	Pressure sores / local infection	No Scar
24	100	0	Symmetrical	N	None	No Scar
25	100	8	Symmetrical	N	Skin erosion/Nail Exposure/ local infection	No Scar
26	100	0	Symmetrical	N	None	No Scar
27	94	4.8	Symmetrical	N	None	No Scar
28	100	0	Non‐Symmetrical	Y	Pressure sores /Functional Limitation of the shoulder joint	No Scar
29	96	4.2	Symmetrical	N	None	No Scar
30	97	1	Symmetrical	N	None	No Scar
31	100	0.8	Symmetrical	N	None	No Scar
32	100	0	Symmetrical	N	Pressure sores	Scar
33	95	3.8	Symmetrical	N	None	No Scar
34	97	5.8	Symmetrical	N	None	No Scar
35	100	0	Symmetrical	N	None	No Scar
36	100	2.5	Symmetrical	N	Pressure sores /protrusion	Scar
37	100	0	Symmetrical	N	None	No Scar
38	100	0	Symmetrical	N	Skin irritation/pressure	No Scar
AVG	98.47368421	1.552631579				
SD	3.802608814	2.184242232				

Footnotes: Y = Yes, N = No, DASH=disabilities of the arm, shoulder and hand
